# Youth well-being predicts later academic success

**DOI:** 10.1038/s41598-022-05780-0

**Published:** 2022-02-08

**Authors:** Diana Cárdenas, Finnian Lattimore, Daniel Steinberg, Katherine J. Reynolds

**Affiliations:** 1 The Australian National University, Canberra, Australia; 2 Gradient Institute, Sydney, Australia; 3 Gradient Institute, Canberra, Australia

**Keywords:** Psychology, Human behaviour

## Abstract

Young people worldwide face new challenges as climate change and complex family structures disrupt societies. These challenges impact on youth’s subjective well-being, with evidence of decline across many countries. While the burden of negative well-being on productivity is widely examined amongst adults, its cost among youth remains understudied. The current research comprehensively investigates the relationship between youth subjective well-being and standardized academic test scores. We use highly controlled machine learning models on a moderately-sized high-school student sample (*N* ~ 3400), with a composite subjective well-being index (composed of depression, anxiety and positive affect), to show that students with greater well-being are more likely to have higher academic scores 7–8 months later (on Numeracy: β* = .033, *p* = .020). This effect emerges while also accounting for previous test scores and other confounding factors. Further analyses with each well-being measure, suggests that youth who experience greater depression have lower academic achievement (Numeracy: β* = − .045, *p* = .013; Reading: β* = − .033, *p* = .028). By quantifying the impact of youth well-being, and in particular of lowering depression, this research highlights its importance for the next generation's health and productivity.

## Introduction

For future prosperity, a nation needs to ensure that the next generation is thriving and developing its productive capability. Despite this, there is concerning evidence that youth subjective well-being is in decline. US adolescents’ life satisfaction, domain satisfaction and happiness have been at a steady decline since 2010 while depression and suicide ideation rates have increased^1,2^. Similar concerning patterns are emerging in Europe^3^, and other countries such as Australia^4^. Given the concerns associated with lower well-being, the aim of this research is to systematically examine the relationship between youth subjective well-being and their academic achievement as an indicator of future productive capacity.

Subjective well-being can be broadly defined as individuals’ beliefs that they have and experience a positive—as opposed to negative—life^5^. It is captured by a sense of satisfaction with one’s life, having positive emotions, and the absence of negative emotions^6^. While these components tend to correlate, subjective well-being is best assessed with multiple indicators^7^.

Declines in well-being have widespread implications for individuals, communities and nations. There are direct costs to the treatment of mental ill-health and also indirect costs to families and the economy through lost productivity and capability. In adulthood, longitudinal and experimental studies show that subjective well-being drives better job performance^8,9^. Critically, the implications of well-being for productivity go beyond individuals. The indirect cost of psychopathological negative affect (e.g., mood disorders such as major depression and anxiety disorders) in Europe is estimated at €798 billion (and $2.5 trillion in the US)^10^, while the Australian Productivity enquiry estimated that improving Australian’s mental health and quality of life would produce financial benefits of approximately $18 billion annually^11^. These analyses go beyond the workplace, quantifying a broader impact on families^12^, communities and the non-government voluntary sector^13^.

Specifically concerning subjective well-being, systematic reviews reveal the association between subjective well-being and performance. Individuals’ emotional affect accounts for 10 to 25% of the variance in job satisfaction^14^. Happy and satisfied individuals consistently perform better in their jobs^15^, show lower turnover^16^, and are more involved in their work life^17^. In addition, individuals who experience positive emotions more often also perform better in work-related tasks as coded by independent observers^18^. Despite strong evidence for the link between subjective well-being and productivity in adulthood, much less is known about youth subjective well-being and its potential consequences for the building blocks of future productivity such as academic achievement.

The limited available evidence suggests a positive association between youth well-being and school performance. For example, students with high subjective well-being are more likely to graduate from college^19^. However, there are challenges and issues with the way this relationship has been examined, making it difficult to quantify the positive impact of youth well-being. A recent systematic review^20^ found that most research tends to be cross-sectional and fails to take into account confounding variables that may simultaneously cause lower subjective well-being and academic achievement such as socioeconomic status and parental education. Without specific quantification, there is a risk that the importance of buffering youth well-being could be disregarded or overlooked.

In this research, we address the methodological limitations in the existing body of work and examine in a systematic fashion whether youth well-being is associated with greater academic performance 7–8 months later. To do so, we draw on recent work in the area of children/youth mental disorders and psychopathology, which tend to use higher-quality methodology (longitudinal, highly controlled models). Results in this area indicate that being diagnosed with a mental disorder or having a high number of its symptoms is associated with worse academic outcomes^21–24^. When standardised test results are used as indicators of performance, there is evidence that children’s (aged 6–11) mental distress, as assessed by their parents, has a negative effect on children’s scores in a national test NAPLAN scores in Australia^25^, the standard academic tests administered by the Australian Curriculum, Assessment and Reporting Authority^26^.

While the existing body of research on psychopathology highlights the achievement drop of highly distressed children, it does not speak to the experience of the great majority of youth who may well experience low subjective well-being without experiencing psychopathology. Moreover, these studies tend to employ methodologies (e.g., medical records; parental assessment of mental distress) that exclude youth’s subjective evaluation of distress. Thus, studies on youth psychopathology do not address questions of youth *subjective* well-being. For this reason, the aim of the current research is to comprehensively examine and *quantify* the relationship between youth subjective well-being and school performance as measured by a standardised test (Numeracy and Reading NAPLAN scores). Specifically, we make use of two waves of data to test whether greater positive affect and lower negative affect (depression and anxiety) predict better performance in a standardised test while controlling for baseline scores on the test and potential confounding factors associated with the student (e.g., age, gender), the family (e.g., parental education) and the schools (e.g., community socio-economic status, staff experience).

Moreover, there is further innovation in the methods adopted in this research. First, a composite subjective well-being score is used that encompasses the presence of positive affect and the absence of negative affect (depression and anxiety). This *subjective well-being index* captures two conceptual subdimensions of the general construct of subjective well-being^5^. The index allows us to examine their *combined* influence of (high) positive and (low) negative affect, which is closer to the theoretical definition of subjective well-being. However, given evidence that positive and negative affect are also different from each other^6^, we also examine how each of the positive and negative affect (depression and anxiety) measures predicts academic achievement. Second, this study makes use of machine learning (ML) models^27^ to test the (statistical) causal association between subjective well-being and academic performance. Machine learning, unlike classical response surface modelling approaches for effect estimation (e.g., ordinary least squares and random effects models), is better able to treat multiple highly-correlated control variables (such as parental education and socioeconomic status)^28–30^. In addition, the statistical flexibility of machine learning permits the modeling of high-dimensional, nonlinear associations among control, treatment, and outcome variables, and, in fact, excels in these conditions^27^. In this research we find a quantifiable benefit in being able to model nonlinear control to treatment, and control to outcome relationships. Machine learning has also successfully been applied to causal inference problems in similar domains^31^.

Quantifying the well-being to performance (i.e., NAPLAN) relationship will help inform whether there needs to be new resources and innovation in the advancement of child and youth well-being. National prosperity with respect to mental health, greater employability, and productive capacity, could depend on such efforts.

## Results

To best estimate an unbiased association between subjective well-being and academic performance, we make use of a large number of controls (40 control variables; a total of 141 after transformation; see Supplementary Table 3). Since standard regression estimators (OLS, hierarchical linear models) are unable to yield effect estimates in these conditions, we use a variety of machine learning models for estimating the effect of subjective well-being on standardised grade 9 test score outcomes (NAPLAN). These models each make different assumptions about the form of the relationships in the data, and have a variety of advantages and disadvantages with regards to estimation bias, as described in the methodology. By using a variety of machine learning models we examine the sensitivity and robustness of the estimated effects to the various modelling assumptions and choices. Broadly speaking, the machine learning models used here can be partitioned into two groups. The first models treat the relationship between the treatment (e.g. well-being index) and the outcome (e.g., NAPLAN score) as linear (linear-in-treatment), but may choose to model the relationship between the controls and treatment or outcome as nonlinear. These are the Bayesian ridge (fully linear), two-stage ridge, and double machine learning (DML) models. For these models we can represent the treatment effect as a standardised regression coefficient (β*). The second group treats *all* relationships as nonlinear, and so we use partial dependence plots^32,33^ to represent the treatment effects for these models. These are the kernelized Bayesian ridge, and gradient boosted tree models.

Estimated effect sizes of the composite well-being index on NAPLAN Numeracy and Reading tests are summarized in Table 1 for the three linear-in-treatment models. Note that the following results should be viewed in light of our structural causal assumptions depicted in Fig. 3, and discussed in the method section. The estimated effect for well-being on Numeracy is statistically significant for all three models (Bayesian ridge, two-stage ridge and DML) at a level-of-significance of α = .05, with greater well-being predicting better Numeracy scores. However, the well-being index did not significantly predict Reading scores in any of the models. Considering the results with respect to NAPLAN scores, the average difference between NAPLAN 7 (a control variable in our model) and 9 Numeracy scores (the dependent variable) in our data is 42.52 points, and the estimated effect of the well-being index from the two-stage model is approximately 2.2 points. So improving an average student’s well-being index by one standard deviation accounts for a ~ 5% (2.2/42.52) improvement in their expected outcomes in grade 9 when controlling for grades 7 NAPLAN scores. The influence of the control variables on the outcome are not reported because the methods employed treat these relationships as “nuisance” quantities that are only estimated as a means-to-an end in creating the best estimate for the treatment-outcome relationship (i.e., well-being-NAPLAN score)^28^. As such, there is no causal interpretation of the controls to outcome relationships.

**Table 1:** Major results for the treatment-outcome effect models (extended results presented in the supplementary material, Table LIN-EXT-RES). β* represents the standardised effect size. Well-being significantly predicts Numeracy outcomes, and depression significantly predicts both Numeracy and Reading outcomes.

Target (grade 9)	Model	N	β* (95% interval)	s.e.(β*) or σ_β*|X, *T*, *Y*_	*p* value	RMSE
**Well-being index treatment**						
Numeracy	Bayesian ridge	3368	0.0294 (0.0092, 0.0495)	0.0103	.0045	39.184
	Two-stage ridge		0.0332 (0.0052, 0.0612)	0.0143	.0202	38.989
	DML		0.0270 (0.0072, 0.0468)	0.0101	.007	NA
Reading	Bayesian ridge	3414	0.0139 (− 0.0086, 0.0364)	0.0115	.2271	45.073
	Two-stage ridge		0.0201 (− 0.0107, 0.0509)	0.0157	.2002	44.475
	DML		0.0189 (− 0.0054, 0.0432)	0.0124	.124	NA
**Self-reported depression treatment**						
Numeracy	Bayesian ridge	3416	− 0.0438 (− 0.0636, − 0.0240)	0.0101	1.5 × 10^–5^	39.412
	Two-stage ridge		− 0.0446 (− 0.0716, − 0.0176)	0.0138	.0012	39.080
	DML		− 0.0475 (− 0.0675, − 0.0275)	0.0102	3 × 10^–6^	NA
Reading	Bayesian ridge	3463	− 0.0385 (− 0.0605, − 0.0165)	0.0112	.0006	45.088
	Two-stage ridge		− 0.0328 (− 0.0622, − 0.0034)	0.0150	.0281	44.556
	DML		− 0.0425 (− 0.0676, − 0.0174)	0.0128	.001	NA

**Figure 1:**
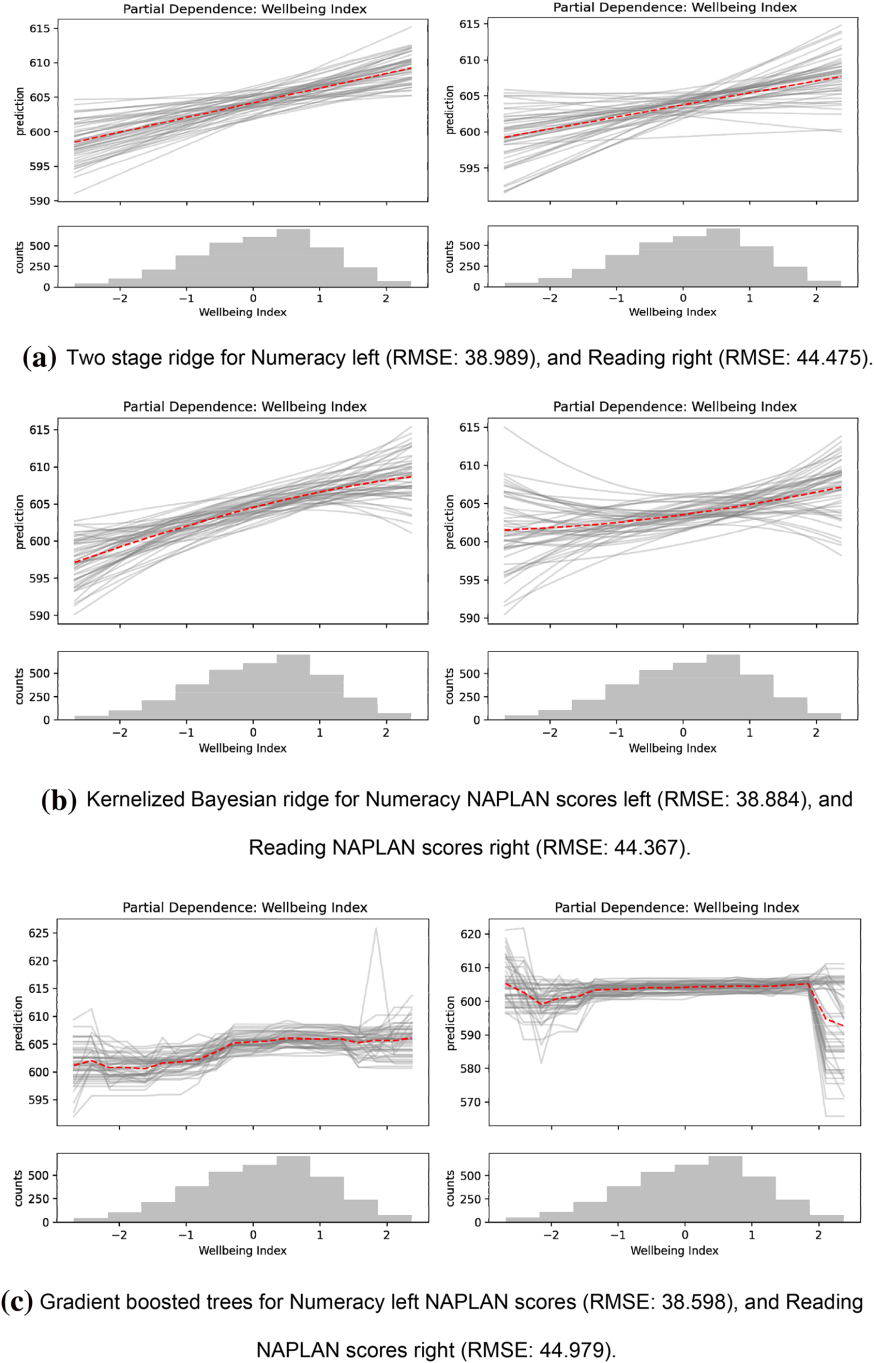
Partial dependence plots of well-being index on NAPLAN *Numeracy* and *Reading* grade 9 scores. These can be interpreted as the (conditional) average treatment effect of well-being index on NAPLAN scores (for year 9 students), i.e. they demonstrate the average effect on NAPLAN of changing a student’s well-being. The histogram beneath each plot represents the density of the treatment variable. Three models have been used here: (**a**) a two stage ridge regressor (linear treatment, kernelized controls), (**b**) an approximate kernelized Bayesian regression (using a Nyström gram matrix approximation), and (**c**) a gradient boosted regression tree. The grey lines are bootstrap samples of the model predictions, and the dashed red line is the mean of the samples. A higher model uncertainty is depicted by less agreement in the gray prediction samples. (**b**) and (**c**) Show mostly linear treatment-outcome effect relationships even though they are completely nonlinear models. These figures were made using Matplotlib (https://matplotlib.org/; ver. 3.3.0).

**Figure 2:**
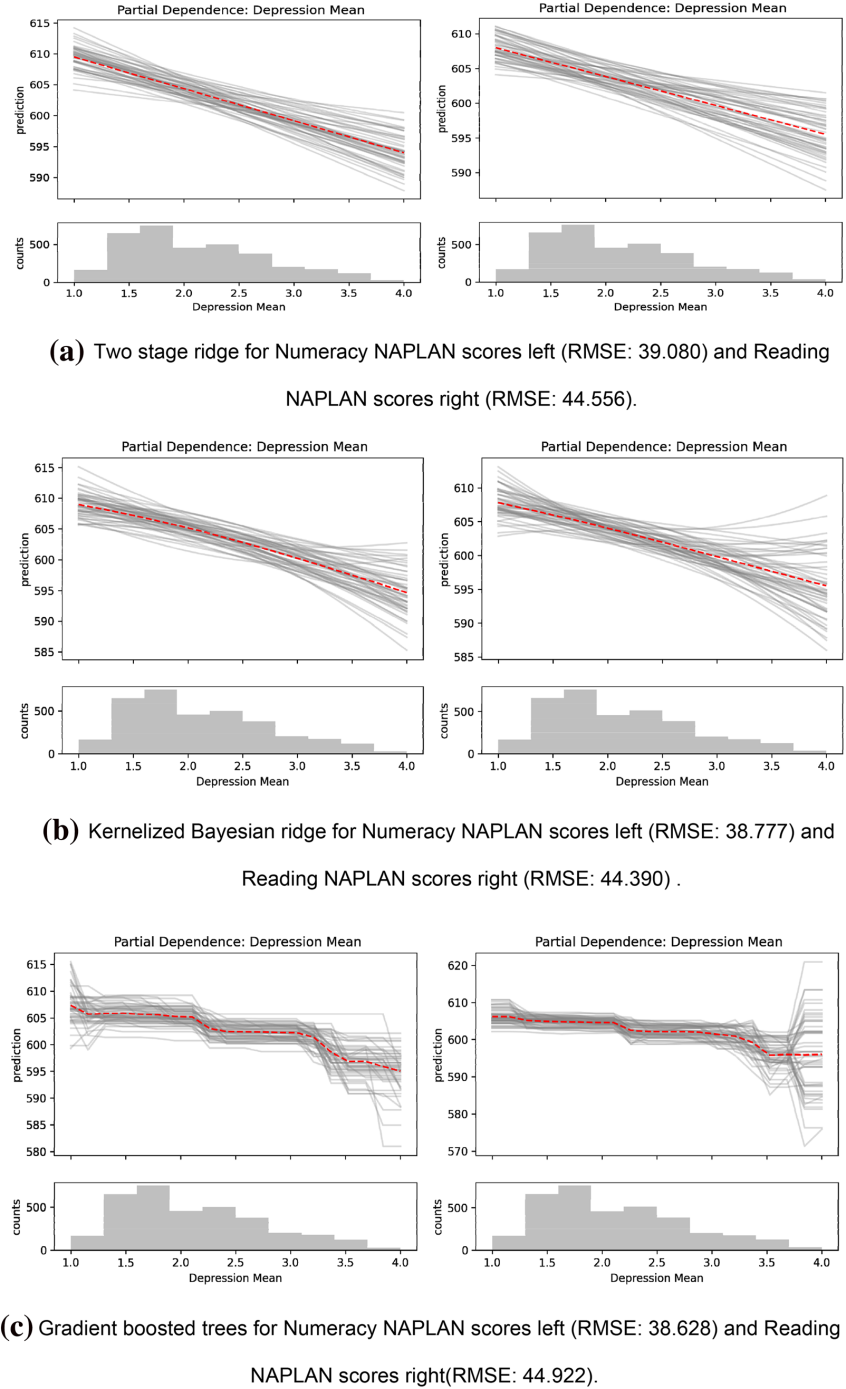
Partial dependence plots of self-reported depression on NAPLAN *Numeracy* and *Reading* grade 9 NAPLAN scores. The description of these plots is the same as those in Fig. 1, but use depression as the treatment variable as opposed to the well-being index. The nonlinear models (**b**) and (**c**) show mostly linear relationships. These figures were made using Matplotlib (https://matplotlib.org/; ver. 3.3.0).

The estimated effects from the two totally nonlinear machine learning models are depicted in Figs. 1 and 2 as partial dependence plots. We also show the linear-in-treatments two-stage ridge regression model in these figures as a point of comparison. Bootstrap average effect estimates in these plots (red dashed line) are relatively linear for both the kernel and tree models, which suggests that the linear-in-treatment models may not suffer from model mis-specification bias. However, it is worth noting that the completely nonlinear and nonlinear-in-controls models slightly, but consistently, outperform the linear Bayesian ridge model—which is completely linear—in terms of cross validation root mean squared error (RMSE). This implies that nonlinear associations exist between the control variables and well-being and/or NAPLAN scores, and therefore completely linear models may be misspecified.

When examining each of the subjective well-being measures independently, self-reported depression negatively and significantly predicted lower Numeracy and Reading NAPLAN scores 7–8 months later. In contrast, self-reported anxiety or positive affect did not significantly predict any NAPLAN score (see Supplementary Table 4 in Supplementary Material). Interestingly, there were indications that, when holding depression as fixed, anxiety may have a small positive effect on NAPLAN scores (though this association was not significant). This may explain why the impact of depression on academic achievement is clearer than that of the well-being index, as this index incorporates self-reported anxiety, depression and positive affect (see Supplementary Fig. 1). In terms of NAPLAN scores, reducing self-reported depression by one standard deviation would increase Numeracy score by 3 points or ~ 7% (3/42.52) and Reading score by 2.5 points, or ~ 7% (2.5/34.80).

## Discussion

Youth well-being trends indicate a decline worldwide, and the potential consequences of this decline on academic performance are not fully understood. The goal of this research is to examine whether youth subjective well-being (a composite score indicating the presence of positive affect and the absence of negative affect, as well as each measure of well-being examined independently) impacts academic performance, which underpins employment prospects and future productivity.

**Figure 3:**
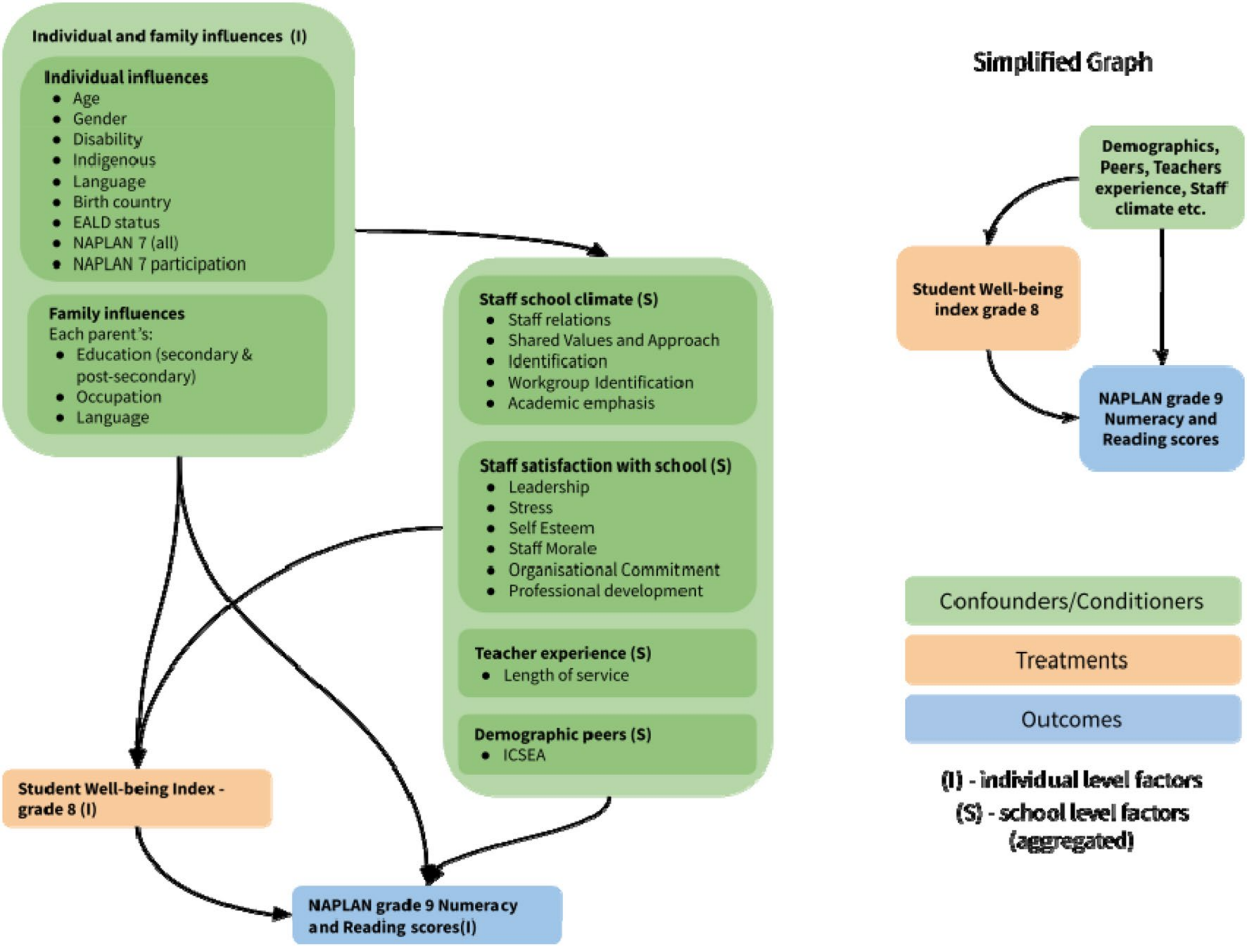
The causal relationships between factors assumed in this study. Some of these factors are at the level of the individual, and others are at the school level. The detailed graph on the left can be simplified into the smaller graph on the right, which was used to inform the modelling approach.

Given the structural causal assumptions depicted in Fig. 3, our modelling results show that subjective well-being predicted greater NAPLAN scores 7–8 months later in models controlling for NAPLAN performance two years prior as well as other key individual, family and school factors (40 confounded variables that, when transformed, result in 141 control variables in the models). This effect was consistent for Numeracy but not for Reading. For every standard deviation increase in subjective well-being, we are likely to observe an increase of two points in Numeracy NAPLAN score. This is important given that NAPLAN results tend to vary one to five points from one year to another^34^. Therefore, a variation of two points in NAPLAN scores represents an important amount of yearly variation.

The results are stronger when we examine the effect of depression only, with every standard deviation decrease in depression predicting an increase of 3 points in Numeracy NAPLAN score. Depression also predicted greater reading scores, with one standard deviation decrease in depression being associated with an increase of approximately 2.5 NAPLAN Reading points. These results suggest that, of the two elements of subjective well-being that we assessed, negative affect, and particularly depressed mood, are of key importance in understanding academic performance. This may be because depression is associated with reduced approach-based goal pursuit motivation^35^, working memory capacity and distraction inhibition^36^. The reduced motivation and cognitive abilities hinder academic achievement. In contrast, anxiety can, under certain circumstances, motivate greater engagement to avoid negative consequences. When individuals have the working memory to engage in the task, this results in higher achievement^37^. Previous studies have found negative^38^ and positive^24^ associations between anxiety and academic achievement. While the current results were null, they illustrate the complex relation between anxiety and academic achievement, as well as the importance of capturing several elements of youth subjective well-being, examining their effect together but also separately. While our results suggest that depression is the primary driver of decreased academic success, we would need a larger sample size to make this claim with confidence.

The hypothesized predictive associations were tested using multiple state-of-the-art machine learning models for causal inference^29,30,39^, which enabled the use of multiple highly correlated covariates as well as non-linear associations. In particular, we designed models to better ‘isolate’ the well-being-academic performance relationship while accounting for confounding variables at the individual (e.g., demographic variables), family (parental education) and school level (e.g., school socioeconomic status, teachers’ perceptions of school climate). Therefore, the use of ML provides assurance that it is subjective well-being that is impacting on academic performance. This pattern of results is observed above and beyond many of the “usual suspects” such as socioeconomic status and parental education. Of importance, we controlled for the NAPLAN score obtained two grades earlier (grade 7 NAPLAN), which allows us to account for two important effects: student’s previously demonstrated ability to achieve, and, since they are in the same school in which they took the previous test, the effects of the same school environment or climate on current school performance.

Another advantage of the ML methods employed is the ability to consider non-linear relations. Despite this, the statistical models provide consistent evidence of a linear association between depression (and more broadly well-being) and academic performance. This suggests that there are no critical levels of depression and well-being more broadly where academic performance will suffer or benefit most. Instead, the results indicate that all school performances would similarly increase with lower depression (and greater well-being), regardless of current level. Altogether, the methodology (subjective well-being predicting NAPLAN scores later in time) and the analytical models employed in this research (ML) provide confidence in the main finding: students that experience greater subjective well-being, particularly those that have lower levels of depression, will be more likely to obtain higher NAPLAN scores.

Given the high proportion of young people that attend (mandatory) primary and secondary schools, schools are well placed to assess and track depression and other well-being indicators. Furthermore, they serve as an institutional site for *liaison* between families and community services, which can together address youth depression and well-being more generally. It is also the case that the ideal school environment, with a positive social climate that fosters a sense of belonging and connection can buffer feelings of depression and promote positive emotions in students^39^ and impact on academic achievement^40^. In addition, school-based depression and anxiety prevention programs have been shown to be effective^41,42^, particularly for students that had high levels of depression^42,43^. Therefore, schools are central to youth well-being.

In this work we thoroughly and robustly quantify the relationship between youth well-being and academic performance. We provide clear and compelling evidence of a relationship between well-being and academic performance, and more precisely, about the role of depression. For the first time, to the best of our knowledge, the impact of youth well-being is quantified on a dimension related to economic opportunity. Nevertheless, despite these strengths, there are certain limitations and opportunities for future research that need to be highlighted. First, a longer period of time between subjective well-being measures and school performance (i.e., more than 7–8 months) would enable the investigation of the long-term effects of subjective well-being. Second, to the best of our knowledge, there has not been a thorough analysis of these machine learning approaches with covariates on multiple levels (e.g. school and individual levels), and so our results should be viewed in light of this fact. While this is an important avenue for future research, it should be noted that our findings are in line with research on youth mental distress^25^. Third, school performance is operationalised as scores in a standardised test. While this test has the advantage of following the Australian curriculum, there are important risks associated with equating school performance with scores on such a test. Performance on these tests represents but one dimension of students'  learning experience and capabilities. As highlighted by Patton and colleagues^44^, equating results of standardised scores with academic performance can shift schools focus towards test scores to the detriment of the wide variety of learning experiences a student undergoes in schools (other subjects, socio-emotional learning, civic education, etc.)^45^. It is also the case that the relationships between standardised test results and employment prospects itself requires some careful examination.

This research provides compelling evidence that promoting youth well-being and students’ current (and future) performance are perfectly synergistic goals. In particular, protecting youth from depression can create a path towards better school performance and its associated benefits for the individual student and national prosperity. Thus, investing in youth well-being “bring[s] benefits today, for decades to come, and for the next generation (p. 2423)”^44^. In so doing, it supports recent and urgent calls to address youth well-being made worldwide^46^.

## Method

### Participants and procedure

In this research, we make use of two primary sources of data. The first is an annual student and staff satisfaction and school climate survey conducted by an Australian state/territory. Students from grade 7 onward respond to a series of questions which include the subjective well-being scales. Staff respond to a similar series of questionnaires intended to gauge their perception of the school environment. Both staff and students provided informed consent before answering these questions. The second source of data is administrative, provided by the school department, which includes student demographic information (e.g., gender, parental education, socioeconomic status), teacher experience information, school socio-educational status (ICSEA)^47^ and our key outcome variables: grade 9 NAPLAN test Numeracy and Reading scores. Administrative data also included the previous NAPLAN score, those of year 7 ( NAPLAN is assessed every two years), which are used as a proxy for prior achievement (i.e., individual ability; the school’s ability to help students achieve, since students remain at the same school in 7th and 9th grade). We make use of subjective well-being measures answered by 8th graders in September 2016 to 2018 and their matched grade 9 NAPLAN scores in May 2017 to 2019. All students’ responses to the annual survey and administrative data are matched at the individual level where feasible, or at the school level where not. The total number of students who had *any* NAPLAN scores from 2017 to 2019 is 7887. Within this cohort of 7887, participation rate for *each* NAPLAN test was just over 80% (see Supplementary Material for more details on missing rates per measure), which is lower than the national average for this time period (90%). NAPLAN tests are optional, as parents can opt out of having their children take any (or all) of the NAPLAN tests. In addition, 50% of the 7887 participants had matching subjective well-being survey data. The survey is optional for students and some schools put greater effort and support than others in facilitating students' response rate.

Once matched, our total sample is composed of N ~ 3400 participants from 19 high schools. Around 2% of students are indigenous, 2% have a recorded disability and a little over 6% are learning English as an additional language. The students are balanced by gender and come from a range of socio-economic backgrounds. The sample varies slightly across the different outcome and treatment measures because of the varying NAPLAN test and survey participation rates. Supplementary Tables 1 and 2 present detailed information on the sample characteristics for each target NAPLAN score. This study complies with all relevant ethical regulations and was approved by the Australian National University human ethics committee.

### Measures

#### Subjective well-being

Subjective well-being was assessed with two measures of negative affect (anxiety and depression) and a measure of positive affect. Anxiety was assessed with the generalized anxiety subscale of the Child Anxiety Related Emotional Disorders measure (SCARED)^48^. The SCARED measures children’s anxiety and has shown good validity and reliability^48,49^. Students rated how often they experienced these emotions and thoughts on a 3-point Likert scale that ranged from 0 (Not true or hardly true) to 2 (True or often true). The scale includes statements such as “I worry about how well I do things” and “I worry about things that happened in the past”. Depression was measured with the Centre for Epidemiological Depression Scale (CES-D) Boston short-form (10 items)^50^. The CES-D measures feelings of depression and has previously demonstrated good internal reliability and content validity when used with adolescents^51,52^. This measure asks students to think about their day-to-day life and indicates how much each statement applied to them (e.g., “I felt lonely”; “I could not get going”). The items were rated on a 5-point Likert scale ranging from 0 (Rarely/none) to 4 (Very often/always). Positive affect was measured with 10 items of the personal well-being subscale of the Australian Adolescent version^53^ of the Mental Health Inventory (MHI)^54^. This scale has good reliability and validity^53,54^ and it measures the number of times that participants experienced positive emotions in the past month using an 8-point scale ranging from 0 (None of the time) to 7 (All of the time). Each of the subjective well-being measures were independently averaged, with an obtained mean for anxiety, depression and positive affect.

In line with previous research using composite well-being scores^55,56^, we standardised the subjective well-being dimensions and created a composite score (using the first principal component of a principal component analysis of these constructs)^57^ that combines positive and the reversed negative affect measures (see Supplementary Material for more details). This was done given a theoretical understanding that positive and negative affect (as well as life satisfaction, which is not measured in this study) represent an underlying construct of general subjective well-being^58,59^. However, given suggestions to treat these components separately^6^, we additionally test the models with each of the individual components.

#### Academic performance

NAPLAN scores are used as a measure of academic performance. NAPLAN is a standardised assessment measuring students’ academic achievement for Numeracy and Reading. The NAPLAN scale ranges from 0 to 1000 score. NAPLAN is administered by the Australian Curriculum, Assessment and Reporting Authority (ACARA) and reflects national curriculum and learning goals in literacy and numeracy. NAPLAN also assesses writing, spelling and grammar, but a recent report review suggests that these subdimensions are generally unreliable (and therefore lack validity)^45^. Therefore, our analyses focus on Numeracy and Reading. NAPLAN is offered to all Australian students in grades 3, 5, 7 and 9.

#### Structural causal assumptions and control variables

In order to estimate the impact of youth well-being on future academic performance it is necessary to consider and adjust for any potential confounding variables that may influence both a student’s well-being in grade 8 and their academic performance in grade 9. Individual covariates were age, sex, disability, Aboriginal self-identification, country of birth, language used at home, whether English was a second language at home, whether they had participated in the previously assessed NAPLAN and their NAPLAN score in 7th grade. In terms of family influences, we adjusted for parental secondary education, parental post-secondary education and parental occupational category. To account for school-related effects on youth subjective well-being and academic outcomes, school socioeconomic status (ICSEA)^47^, staff’s perceptions of the school environment (school climate)^39^, their school satisfaction^60^ and teacher experience were all controlled. These structural assumptions are depicted in Fig. 3. The final number of control variables used was 40, which became 141 when we encoded dummy and missing values.

### Estimation methodology

Since we have over 141 factors to control for (after dummy missing value encoding), many of which are highly correlated, we cannot resort to classical observational methods based on ordinary least squares (OLS) or unregularised hierarchical modelling (HM) to infer our treatment effects. Furthermore, we could not establish *a-priori* whether the relation between subjective well-being and academic performance is linear. For instance, it is possible that very low subjective well-being is particularly detrimental to academic performance (as observed in the mental disorders literature) but that this relationship becomes less pronounced at higher levels of subjective well-being. Therefore, we use machine learning methods for our analysis, as they can model nonlinear relationships and can perform inference effectively in high-dimensional settings^27^. Broadly, these methods assume that the high dimensional and non-linear relationships between the control variables and the treatment/outcome variables are “nuisance” relationships and are only included to ensure the treatment-outcome relationship is unconfounded^28^. This assumption allows us to use black-box machine learning models to learn these complex nuisance relationships, while freeing us to explicitly parameterise the treatment-outcome relationship if deemed necessary.

The most straight-forward application of machine learning to observational causal inference is *direct response surface modelling* (DRSM) as described by Hill^30^. This amounts to using machine learning models to regress the control variables and treatment on the outcome. Since machine learning models can represent a wide variety of nonlinear relationships, this approach has the advantage of reducing the likelihood of introducing bias into the estimation of treatment effect due to model mis-specification. However, to function in high dimensional settings (and not “overfit” the data), many machine learning models use parameter regularisation (or model complexity penalty).

This regularisation may have the unfortunate side-effect of introducing bias into treatment effect estimation by either introducing confounding^61^, or suppressing the treatment-outcome relationship. To rectify this issue, *double machine learning* (DML)^28,61,62^ and *two-stage ridge* (TS) regression methods^61^ have been developed. These allow for treatment effect inference to be performed in the presence of high-dimensional and nonlinearly related control variables with minimal bias from regularisation. Unfortunately, research in this area has been mostly limited to linear treatment-outcome relationships, and so may be susceptible to model mis-specification bias. All of these methods are compared in Table 2. We make use of DRSM, DML and TS methods as described in the next section as a form of sensitivity analysis to establish how robust the treatment effect estimate is to our choice of modelling approach. However, this is an emerging field, and there are few implementations of these methods (software) that support continuous treatment variables available at the time of publication.

**Table 2:** A comparison of machine learning approaches to observational causal inference.

Method	Susceptible to regularisation bias	Susceptible to model mis-specification bias	Readily available software
Direct response surface modelling (DRSM)	More	Less	Y
Two-stage ridge (TS)	Less	More	N (but this was easily implemented)^a^
Double machine learning (DML)	Less	More	Y

^a^The code for this can be found at https://github.com/gradientinstitute/twostageridge.

Reporting treatment effects in a consistent and comparable manner for these machine learning methodologies presents a challenge. Linear treatment-outcome effect relationships can be reported as a standardised regression coefficient, β*, that represents the standardised average treatment effect (ATE) of changing the treatment by one standardised unit on the outcome in standardised units (β* can also be used to represent the conditional average treatment effect [CATE] by subsetting the data. Our method for representing nonlinear effect relationships can be similarly used to represent the CATE). However, since most of our linear models make use of some form of regularisation, these estimates of β* may incorporate some level of bias (though less so for two-stage and double machine learning models). For nonlinear treatment-outcome relationships, representing the ATE is less straightforward since a standardised coefficient is no longer sufficient. We now present our method for representing nonlinear (and linear) ATE by first introducing some notation.

We use N to represent the number of participants in our analysis, and *i* to denote the index of an individual in the data. $Y \in {\mathbb{R}}$ is the outcome random variable (dependent variable), and is an unknown function of the control variables and the treatment variables. An instance of this random variable is denoted as *y*. ${\mathbf{X}}$ is a vector of the control random variables, for simplicity of exposition we will represent these as in ${\mathbb{R}}^{{\text{D}}}$ (D being the dimensionality of ${\mathbf{X}}$), but in reality they are in a more general set, χ that includes categorical and real valued numbers. An instance is denoted as ${\mathbf{x}}$. $T \in {\mathbb{R}}$ is the treatment variable, which is influenced by a (subset) of the control variables, and also influences the outcome variable. An instance is denoted as t. ${\text{f}}({\mathbf{x}},t):{\mathbb{R}}^{{\text{D}}} \times {\mathbb{R}} \to {\mathbb{R}}$ is a (machine learning based) regression estimate of E[*Y* | **X**, *T*] that maps **X** and T to outcomes, Y. Given the structural assumptions before, the average treatment effect (ATE) of well-being (*T*) on NAPLAN (*Y*) is;

$${\mathbb{E}}\left[ {Y|{\text{do}}\left( {T = t} \right)} \right] = \int_{{{\mathbb{R}}^{D} }} {{\mathbb{E}}\left[ {Y|{\mathbf{X}} = {\mathbf{x}},{\text{do}}\left( {T = t} \right)} \right]} p\!\left( {{\mathbf{X}} = {\mathbf{x}}} \right){\text{d}}{\mathbf{x}}{.}$$

here the notation do(*T* = *t*) represents an exogenous intervention that sets the treatment variable *T* to some value *t*^63^, and *p*(⋅) is a probability density function. We estimate this quantity via a plugin estimator, f(**x**, *t*), using machine learning regression models’ estimate of ${\mathbb{E}}\left[ {Y|{\mathbf{X}} = {\mathbf{x}},T = t} \right]$ to give,

$${\mathbb{E}}\left[ {Y|{\text{do}}\left( {T = t} \right)} \right] \approx \frac{1}{N}\sum\limits_{i = 1}^{N} {f\left( {{\mathbf{x}}_{i} ,t} \right).}$$

This is the same quantity estimated by *partial dependence* (PD) plots^32,64^, and so we use PD plots to estimate ATE for a sweep through the available treatment levels, *t*, present in the data as per Zhao^32,33^, see Figs. 1 and 2 for an example. For a linear model it can be straightforwardly shown that this expression simplifies to β⋅*t* + c, for a regression coefficient β and some constant c.

It is important to quantify the uncertainty of the estimated ATE. For linear models that can parametrize ATE as an unregularised regression coefficient—such as the two-stage model^61^—we use the OLS finite sample estimate of standard error, s.e.(β). We assume the degrees of freedom (d.f.) for this statistic is N—D, where D is the number of covariates including the intercept input into the model. This assumption is conservative since the other regression coefficients are regularised, which lowers the effective D, and so we may compute a higher level of estimated uncertainty for β. To obtain a standardised variant, s.e.(β*), we divide s.e.(β) by the sample standard deviation s_*Y*_. For testing the significance of β in these models we use a two-sided t-test with the statistic τ_N−D_ = β/s.e.(β).

We also use a Bayesian linear model that provides a posterior distribution over β directly( see Tipping^65^ for its parameterisation and inference algorithm). This model is implemented in scikit-learn^66^ as the BayesianRidge class (https://scikit-learn.org/stable/modules/generated/sklearn.linear_model.BayesianRidge.html), and we use its default hyperprior settings. We use this model’s posterior standard deviation, σ_β|**X**,*T*,*Y*_, to construct the t-test statistic τ_N−D_ = β/σ_β|**X**,*T*,*Y*_ following Halawa^64^. This test will also be biased from estimating d.f. ≈ N − D and from the shrinkage prior over β, but again it will be conservative in that it over-estimates uncertainty compared to an OLS estimator. We also obtain a standardised σ_β*|**X**,*T*,*Y*_ by dividing by s_*Y*_.

For visualising the uncertainty in the nonlinear ATE estimators, we use bootstrap resampling^67^. Specifically, we randomly resample the data with replacement to train the regression model and to compute the PD plot. This is repeated 50 times for each regression model. Each random PD plot sample (grey curves) and their mean (red curve) is then drawn in the plot (see Figs. 1, 2). The less agreement there is in the PD plot samples indicates an increased model uncertainty in the ATE estimate. We have not quantified the uncertainty from these estimators by, for example, constructing confidence intervals, since this would have required many more bootstrap samples and would have been computationally intensive. We viewed this extra step as unnecessary since we obtained quantified uncertainty measures from the linear-in-treatment models, and these nonlinear models did not show much evidence for highly nonlinear treatment-outcome relationships.

These uncertainty estimates are *only* of the machine learning regression model *parameters* since the hyperparameters were fixed for training on the randomly resampled training data (except for the Bayesian ridge regression model, which uses a two-level hierarchical prior that was learned using empirical Bayes methods, and so did not require cross-validated model selection; however, we still obtained model RMSE using this cross-validation procedure). The hyperparameter selection procedure used for all machine learning models was a grid search using stratified K-Fold cross validation^66^, where the folds were stratified with respect to schools. This stratification was required to ensure the models did not overfit on the school-level values, and so bias the out-of-fold error estimates. This hyperparameter selection procedure is where we obtained the model root mean squared error (RMSE) and was conducted before the bootstrap resampling procedure to quantify ATE estimation uncertainty.

Individuals who had data missing in the depression, anxiety or positive affect constructs were excluded from the analysis. For those with missing data in the control variable data, their data was mean imputed for continuous attributes, and a new missing category was assigned for their categorical attributes. A missing dummy variable was also created for continuous control variables that gave the machine learning estimators extra information to help compensate for the missing information. This imputation approach when used in conjunction with bootstrap resampling yields a simplified multiple imputation strategy^68^ for the purposes of creating the PD plot estimates of ATE. See Supplementary Table 3 for statistics on the proportion of missing data in our controls for the well-being index treatment.

### Models

The following equations describe the basic model forms of each of the machine learning estimators used in this analysis. We assume for simplicity that the control variables, **x**, include a “1” element that represents an intercept term. We use the python library scikit-learn^66^ for the implementations of all of our models apart from the two-stage ridge regression model (which we implemented in pure python) and the DML models, which use the EconML library^69^.

### Bayesian ridge regression (DRSM)

$$y =\upbeta \cdot t + {\mathbf{x}}^{{\text{T}}} {{\varvec{\upgamma}}} + \upepsilon \quad \quad \left( {\upbeta ,{{\varvec{\upgamma}}}} \right)\sim{\text{N}}\left( {{{\varvec{\upmu}}},{{\varvec{\Sigma}}}} \right),\;\;\upepsilon \sim{\text{N}}\left( {0,\upsigma _{\upepsilon }^{2} } \right)$$

With **μ** and **Σ** given by Eqs. (12) and (13) in^65^. This model uses type-II maximum likelihood to learn the prior distribution parameters. See scikit-learn’s “BayesianRidge” model^66^ for implementation specifics.

### Bayesian kernelized regression (DRSM)

$$y = {\upvarphi }\left( {{\mathbf{x}},t} \right)^{{\text{T}}} {{\varvec{\upgamma}}} + \upepsilon \quad \quad {{\varvec{\upgamma}}}\sim{\text{N}}\left( {{{\varvec{\upmu}}},{{\varvec{\Sigma}}}} \right),\;\;\upepsilon \sim{\text{N}}\left( {0,\upsigma _{\upepsilon }^{2} } \right)$$

where φ(**x**, t) is a Nyström Kernel function basis approximation^70^. A radial basis kernel was used, with cross validation as described previously to choose the length-scale. Again, **μ** and **Σ** given by Eqs. (12) and (13) in^65^ with type-II maximum likelihood learning of the prior parameters. See scikit-learn’s “Nystroem” transform and “BayesianRidge” model^66^ for implementation specifics.

### Two-stage kernelized ridge regression (TS)

$$\begin{array}{*{20}l} {{\text{t}} = {\upvarphi }\left( {\mathbf{x}} \right)^{{\text{T}}}\varvec{\updelta} +\upnu } \hfill & {\upnu \sim{\text{N}}\left( {0,\upsigma_\upnu ^{2} } \right)} \hfill \\ {y =\upbeta \cdot (t - {\upvarphi }\left( {\mathbf{x}} \right)^{{\text{T}}} {{\varvec{\updelta}}}) + {\upvarphi }\left( {\mathbf{x}} \right)^{{\text{T}}} {{\varvec{\upgamma}}} +\upepsilon } \hfill & {\upepsilon \sim{\text{N}}\left( {0,\upsigma _{\upepsilon }^{2} } \right)} \hfill \\ \end{array}$$

where φ(**x**) is a Nyström Kernel function basis approximation^70^ with a radial basis function kernel that is the same in both estimation stages. Both of these stages have l_2_ regularisers applied to the weights, **δ** and **γ**, and so are ridge regression estimators. The coefficient β is unregularised, and this formulation reduces the bias induced from applying regularisation to the other model weights, see Hahn and colleagues^29^, for more information on this model. Note that this model assumes a linear treatment-outcome relationship.

### Gradient boosted trees (DRSM)

$$\begin{aligned} y & = {\text{h}}\left( {{\mathbf{x}},t;{\mathbf{r}}_{1} ,{\mathbf{d}}_{1} } \right) + {\text{h}}\left( {{\mathbf{x}},t;{\mathbf{r}}_{2} ,{\mathbf{d}}_{2} } \right) + \cdots + {\text{h}}\left( {{\mathbf{x}},t;{\mathbf{r}}_{{\text{K}}} ,{\mathbf{d}}_{{\text{K}}} } \right) +\upepsilon \\\upepsilon & \sim{\text{N}}(0,\upsigma _{\upepsilon }^{2} ) \\ \end{aligned}$$

where h(⋅,⋅; **r**, **d**) is a decision tree with root nodes **r** and decision rules **d**. Each of the K decision trees is fit to the data successively on the residuals from the last sum-of-trees fit^30,71^. The number of trees, the depth of the trees, and the learning rate were all chosen using the previously described cross validation procedure. See scikit-learn’s^66^ Pedregosa model^66^ for implementation specifics.

### EconML (DML)

$$\begin{array}{*{20}l} {t = {\text{g}}\left( {{\mathbf{x}}_{{\mathbf{1}}} ,{\mathbf{x}}_{{\mathbf{2}}} } \right) +\upnu } \hfill & {{\text{E}}\left[ {\upnu |{\mathbf{x}}_{{\mathbf{1}}} ,{\mathbf{x}}_{{\mathbf{2}}} } \right] = 0} \hfill \\ {y =\upbeta ({\mathbf{x}}_{{\mathbf{1}}} ) \cdot \left( {t - {\text{g}}\left( {{\mathbf{x}}_{{\mathbf{1}}} ,{\mathbf{x}}_{{\mathbf{2}}} } \right)} \right) + {\text{h}}\left( {{\mathbf{x}}_{{\mathbf{1}}} ,{\mathbf{x}}_{{\mathbf{2}}} } \right) + \upepsilon } \hfill & {{\text{E}}\left[ {\upepsilon |{\mathbf{x}}_{{\mathbf{1}}} ,{\mathbf{x}}_{{\mathbf{2}}} } \right] = 0} \hfill \\ \end{array}$$

Here **x** has been split into two sets, **x**_**1**_ and **x**_**2**_, where **x**_**2**_ are the control variables, and **x**_**1**_ are variables that we wish to condition the treatment effect on to model CATE. **x**_**1**_ can also be in the set of control variables. g(⋅,⋅), h(⋅, ⋅) and β(⋅) are all potentially nonlinear functions, and so we can recognise DML as a generalisation of the two-stage ridge regression model to directly allow for modelling of a nonlinear CATE w.r.t. **x**_**1**_ given by the function β(**x**_**1**_)^62^. Note however, that relationship $t \to y$ is still linear. Our particular implementation of DML uses gradient boosting regressors for g(⋅,⋅) and h(⋅, ⋅), with a linear last stage. For this we used the EconML “LinearDML” model^69^.

## Supplementary Information

Supplementary Information.

## Data Availability

The data for this research cannot be made publicly available as it belongs to an Australian education jurisdiction. However, the corresponding author is available to answer any queries concerning the data.

## Code availability

The code used in these analyses is available at https://github.com/gradientinstitute/twostageridge.
