# Supplementary material for: Youth well-being predicts later academic success
Source: Sci Rep. 2022 Feb 8;12:2134. doi: 10.1038/s41598-022-05780-0 (PMC8826920; doi:10.1038/s41598-022-05780-0)
Supplement: Supplementary file 1 — Supplementary Information. [file 41598_2022_5780_MOESM1_ESM.doc]

### Supplementary Material

#### Survey and NAPLAN participation rates

The total number of students who had any NAPLAN scores from 2017-2019 is 7887. Within this cohort about 50% had matching subjective well-being survey data,

Anxiety 50.5 %

Positive Affect 50.6 %

Depression 49.9 %

Well-being Index 49.2 %

Out of this cohort of 7887, many students were also missing one or more NAPLAN score, the exact participation rates for each NAPLAN test between 2017 and 2019 is,

NAPLAN Reading 82.8 %

NAPLAN Writing 83.3 %

NAPLAN Spelling 82.6 %

NAPLAN Grammar 82.6 %

NAPLAN Numeracy 81.2 %

#### Data

**Supplementary Table 1.** Participant information on sample size and individual characteristics.

|  | **NAPLAN Numeracy Score** | **NAPLAN Reading Score** |
| --- | --- | --- |
| **Sample size** | 3404 | 3451 |
| **Number of Schools** | 19 | 19 |
| **Age *M* (*SD*)** | 14.58 (0.36) | 14.58 (0.36) |
| **% Indigenous status** | 2.3 | 2.4 |
| **% EALD** | 6.5 | 6.4 |
| **% Females (Other)** | 50.15 (0.03) | 50.13 (0.03) |
| **% Disability** | 2.1 | 2.3 |
| **NAPLAN score in 7th grade *M* (*SD*)** | 566.81 (69.22) | 573.31 (72.15) |
| **% Participated in NAPLAN in 7th grade** | 89.3 | 89.8 |
| **School level Socioeconomic *M* (*SD*)** | 1086.24 (52.23) | 1086.11 (52.40) |

Notes. EALD = English as an Additional Language or Dialect .

**Supplementary Table 2.** Participant information on family characteristics.

|  |  | **NAPLAN Numeracy Score** | **NAPLAN Reading Score** |
| --- | --- | --- | --- |
| **Lowest parental school education (%)** | **Year 9 or below** | 2.9 | 2.9 |
|  | **Year 10** | 13.3 | 13.4 |
|  | **Year 11** | 4.8 | 4.7 |
|  | **Year 12** | 66.7 | 66.5 |
|  | **Unknown** | 12.4 | 12.5 |
| **Highest parental school education (%)** | **Year 9 or below** | 1.1 | 1.1 |
|  | **Year 10** | 5.3 | 5.3 |
|  | **Year 11** | 2.5 | 2.5 |
|  | **Year 12** | 78.7 | 78.6 |
|  | **Unknown** | 12.4 | 12.5 |
| **Lowest parental non-school education (%)** | **No non-school education** | 16.0 | 16.3 |
|  | **Certificate I to IV** | 20.9 | 20.6 |
|  | **Advanced diploma/Diploma** | 13.1 | 13.3 |
|  | **Bachelor degree or above** | 36.8 | 36.4 |
|  | **Unknown** | 13.2 | 13.3 |
| **Highest parental non-school education (%)** | **No non-school education** | 7.0 | 7.3 |
|  | **Certificate I to IV** | 14.7 | 14.3 |
|  | **Advanced diploma/Diploma** | 11.6 | 11.7 |
|  | **Bachelor degree or above** | 53.5 | 53.4 |
|  | **Unknown** | 13.2 | 13.3 |
| **Lowest parental occupation (%)** | **Not in paid work** | 13.1 | 13.1 |
|  | **Hospitality, assistants & labourers** | 8.6 | 8.6 |
|  | **Trade, sales & service** | 18.4 | 18.4 |
|  | **Management/associate professional** | 15.5 | 15.3 |
|  | **Senior management/professional** | 18.6 | 18.7 |
|  | **Unknown** | 25.8 | 25.9 |
| **Highest parental occupation (%)** | **Not in paid work** | 3.0 | 3.1 |
|  | **Hospitality, assistants & labourers** | 3.7 | 3.6 |
|  | **Trade, sales & service** | 11.1 | 11.1 |
|  | **Management/associate professional** | 16.7 | 16.6 |
|  | **Senior management/professional** | 39.7 | 39.6 |
|  | **Unknown** | 25.8 | 25.9 |

**Supplementary Table 3.** Participant missing data proportions for the well-being index treatment.

| **Variable** | **Missingness proportion** | **Variable** | **Missingness proportion.** |
| --- | --- | --- | --- |
| ParentMAX_Occ_Code | 0.000 | gender | 0.000 |
| ParentMIN_nonSchl_Educ_Code | 0.000 | NAPLAN_ReadingScore_7 | 0.124 |
| StudentLangCode | 0.000 | disability | 0.000 |
| BirthCountryCode | 0.000 | NAPLAN_SpellingScore_7 | 0.122 |
| ReadingParticipation_7 | 0.076 | ParentMIN_Schl_Educ_Code | 0.000 |
| staff_Staff_Morale_Mean | 0.000 | ICSEA | 0.000 |
| NAPLAN_WritingScore_7 | 0.122 | ParentBLangCode | 0.000 |
| NAPLAN_NumeracyScore_7 | 0.131 | Indigenous | 0.000 |
| LanguageConvParticipation_7 | 0.076 | NAPLAN_GP_Score_7 | 0.122 |
| staff_Staff-Student_Relations_Mean | 0.000 | staff_Staff_Relations_Mean | 0.000 |
| staff_School_Identification_Mean | 0.000 | ParentMAX_nonSchl_Educ_Code | 0.000 |
| EALD_Status | 0.000 | Age@30April | 0.000 |
| Wellbeing Index | 0.000 | staff_Organisational_Commitment_Mean | 0.000 |
| staff_Leadership_Mean | 0.000 | ParentMAX_Schl_Educ_Code | 0.000 |
| staff_Self-Esteem_Mean | 0.000 | staff_Shared_Values_and_Approach_Mean | 0.000 |
| NumeracyParticipation_7 | 0.076 | WritingParticipation_7 | 0.076 |
| ParentALangCode | 0.000 | teacher_Length_of_Service_at_end_of_Year_q0.5 | 0.000 |
| ParentMIN_Occ_Code | 0.000 | staff_Academic_Emphasis_Mean | 0.000 |
| staff_Professional_Development_Mean | 0.00 | staff_Work_Group_Identification_Mean | 0.000 |

#### Extended results

**Supplementary Table 4: Extended results for the linear models using self-reported anxiety and positive affect as treatment factors.**

| **Target (grade 9)** | **Model** | **N** | **β* (95% interval)** | **s.e.(β*) or σβ*|X, *_T_*, *_Y_*** | **p-value** | **RMSE** |
| --- | --- | --- | --- | --- | --- | --- |
| **Self-reported anxiety treatment** | | | | | | |
| Numeracy | Bayesian ridge | 3450 | -0.0038 (-0.0242, 0.0166) | 0.0104 | 0.7160 | 39.324 |
|  | Two-stage ridge |  | -0.0133 (-0.0405, 0.0139) | 0.0139 | 0.3381 | 38.896 |
|  | DML |  | -0.0026 (-0.0228, 0.0176) | 0.0103 | 0.804 | NA |
| Reading | Bayesian ridge | 3501 | 0.0110 (-0.0117, 0.0337) | 0.0116 | 0.3404 | 44.860 |
|  | Two-stage ridge |  | 0.0060 (-0.0242, 0.0362) | 0.0154 | 0.6984 | 44.481 |
|  | DML |  | 0.0099 (-0.0130, 0.0328) | 0.0117 | 0.399 | NA |
| **Self-reported positive affect treatment** | | | | | | |
| Numeracy | Bayesian ridge | 3457 | 0.0137 (-0.0061, 0.0335) | 0.0101 | 0.1723 | 39.195 |
|  | Two-stage ridge |  | 0.0107 (-0.0167, 0.0381) | 0.0140 | 0.4434 | 38.900 |
|  | DML |  | 0.0108 (-0.0092, 0.0308) | 0.0102 | 0.29 | NA |
| Reading | Bayesian ridge | 3508 | -0.0015 (-0.0235, 0.0205) | 0.0112 | 0.8947 | 44.986 |
|  | Two-stage ridge |  | 0.0103 (-0.0403, 0.0197) | 0.0153 | 0.5015 | 44.433 |
|  | DML |  | 0.0014 (-0.0247, 0.0219) | 0.0119 | 0.908 | NA |


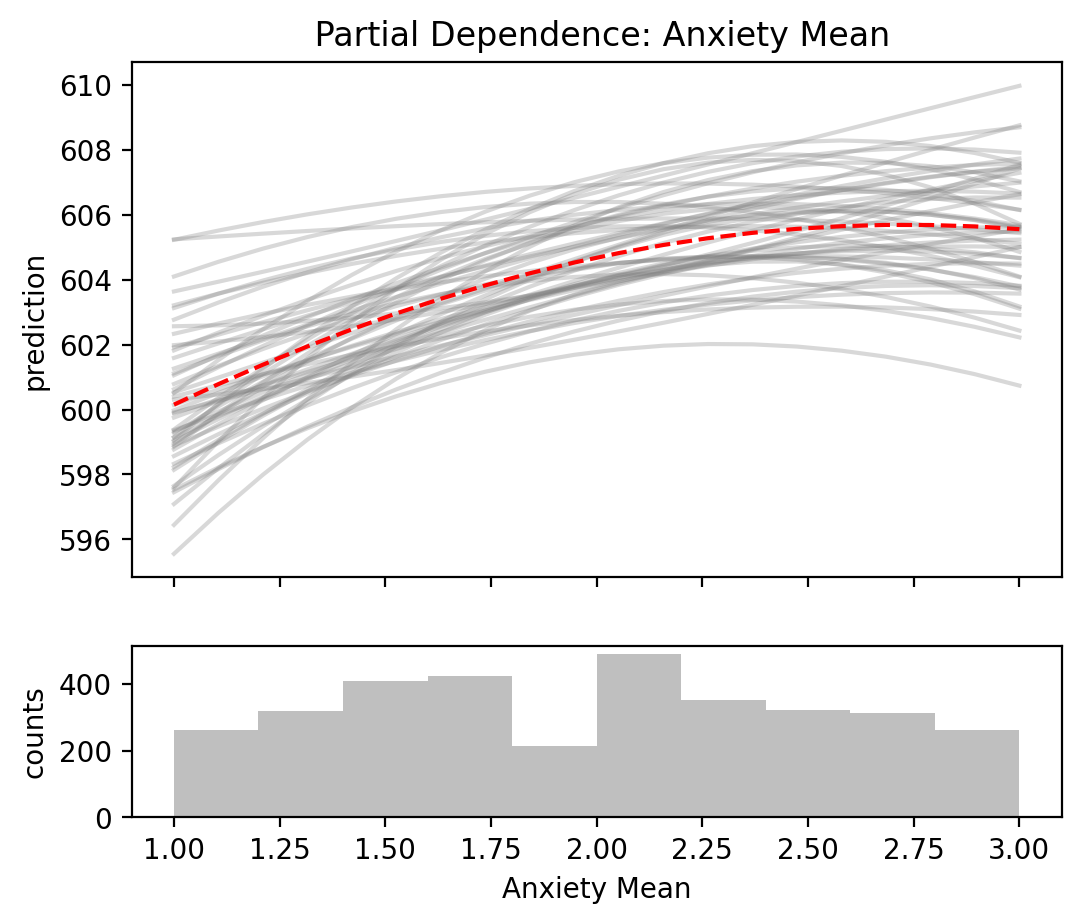

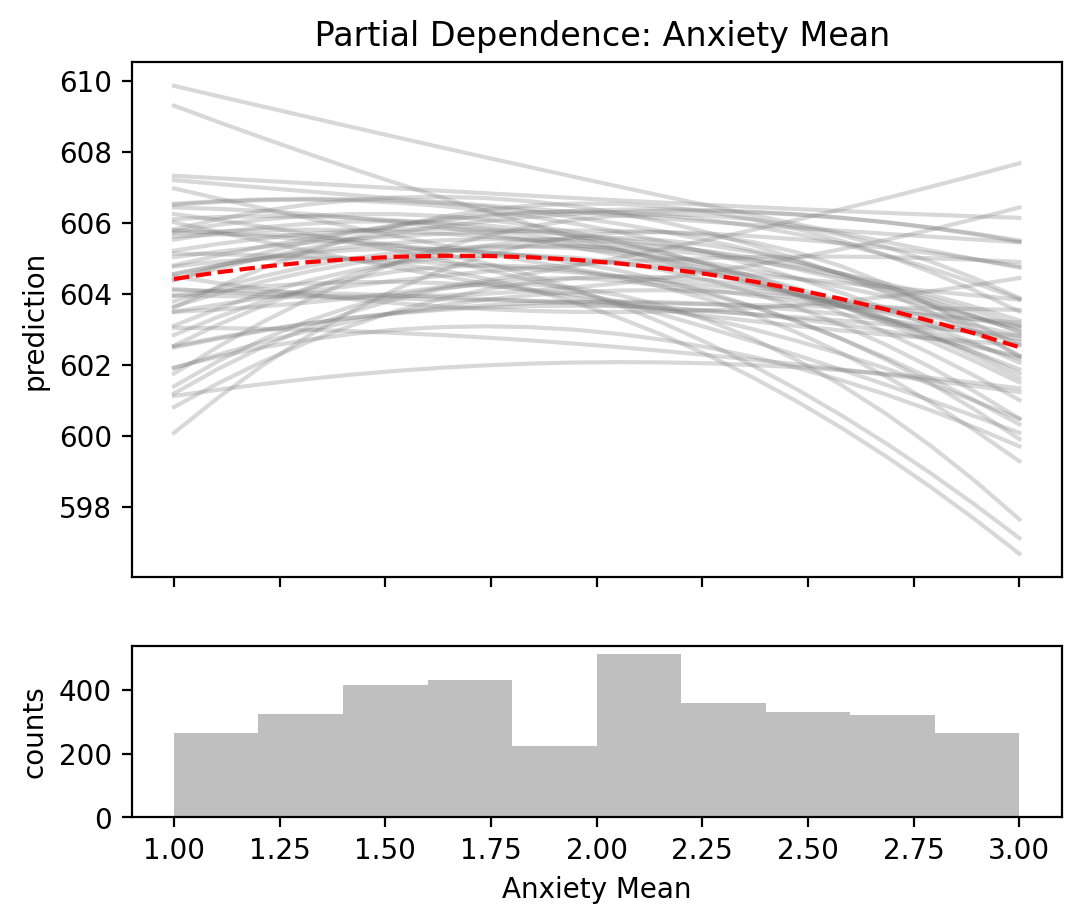


**Supplementary Figure 1**: Reversal of treatment effect from self-reported anxiety on NAPLAN Numeracy score when adding self-reported depression to the kernalized Bayesian ridge model controls (left). The treatment effect from self-reported anxiety on NAPLAN numeracy score without depression in the controls (right). Neither of these effects are statistically significant, however many of the models used show a similar trend. These figures were made using Matplotlib (<https://matplotlib.org/>; ver. 3.3.0).

#### Subjective well-being composite score

Given the theoretical understanding that positive and negative affect represent an underlying construct of general subjective well-being (e.g., Pavot & Diener, 2008;Tov & Diener, 2007), as well as the high intercorrelation between the different subjective well-being measures (see Supplementary Figure 1), a composite subjective well-being score was created. The goal of this composite measure is to capture the most salient information about youth’s subjective well-being from these three existing constructs. Under the assumption that these constructs are linearly related, the optimal method of combining them into one factor such that co-variation is maximally preserved is to use the first principal component of a principal component analysis (PCA) of these constructs (Hotelling, 1933). Running PCA on the full student climate dataset for students for whom these three constructs were present (N = 31981 students from grades 7 to 12 in years 2016-19) resulted in the analysis depicted in Figure 4. Wwe standardised their values before feeding the anxiety, depression and positive affect construct means into PCA . We then used the standardised first principal component of this PCA as the “well-being index” (WBI) that is the primary treatment variable in our analysis. Its unstandardised form is given below,

WBI ≈ 0.51 ༝ standardised positive affect mean

- 0.58 ༝ standardised anxiety mean
 - 0.64 ༝ standardised depression mean.

WBI explains more than 50% of the variation in the positive affect, anxiety and depression constructs among the student cohort, with an increasing WBI value indicating greater well-being.


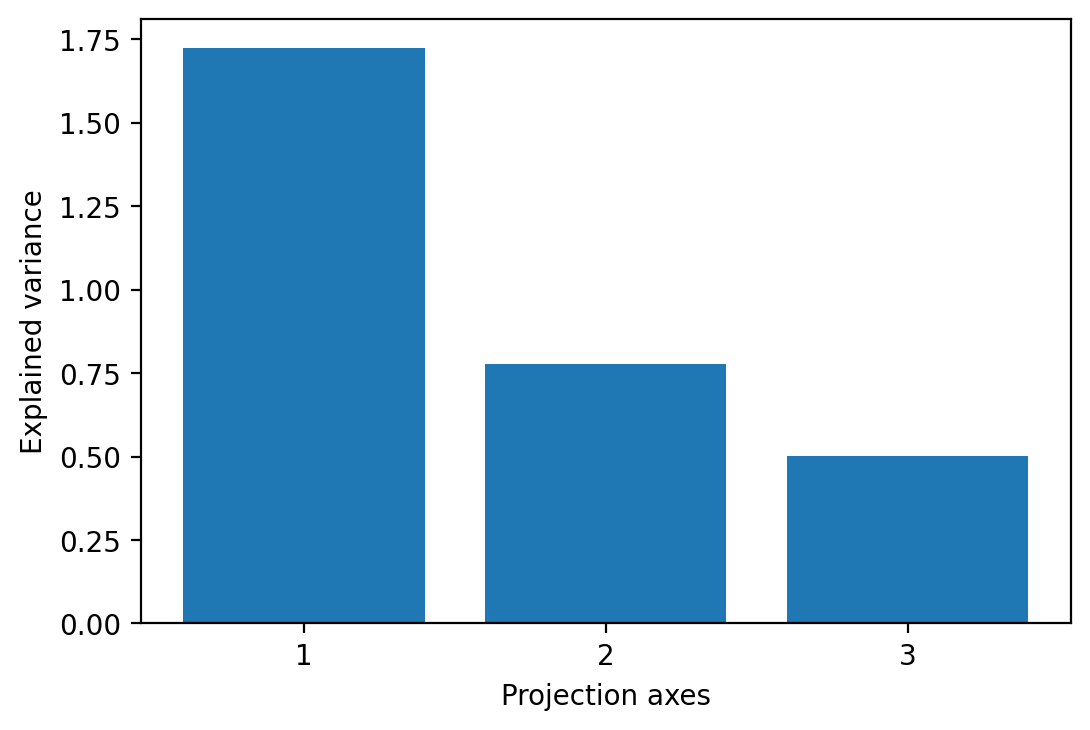

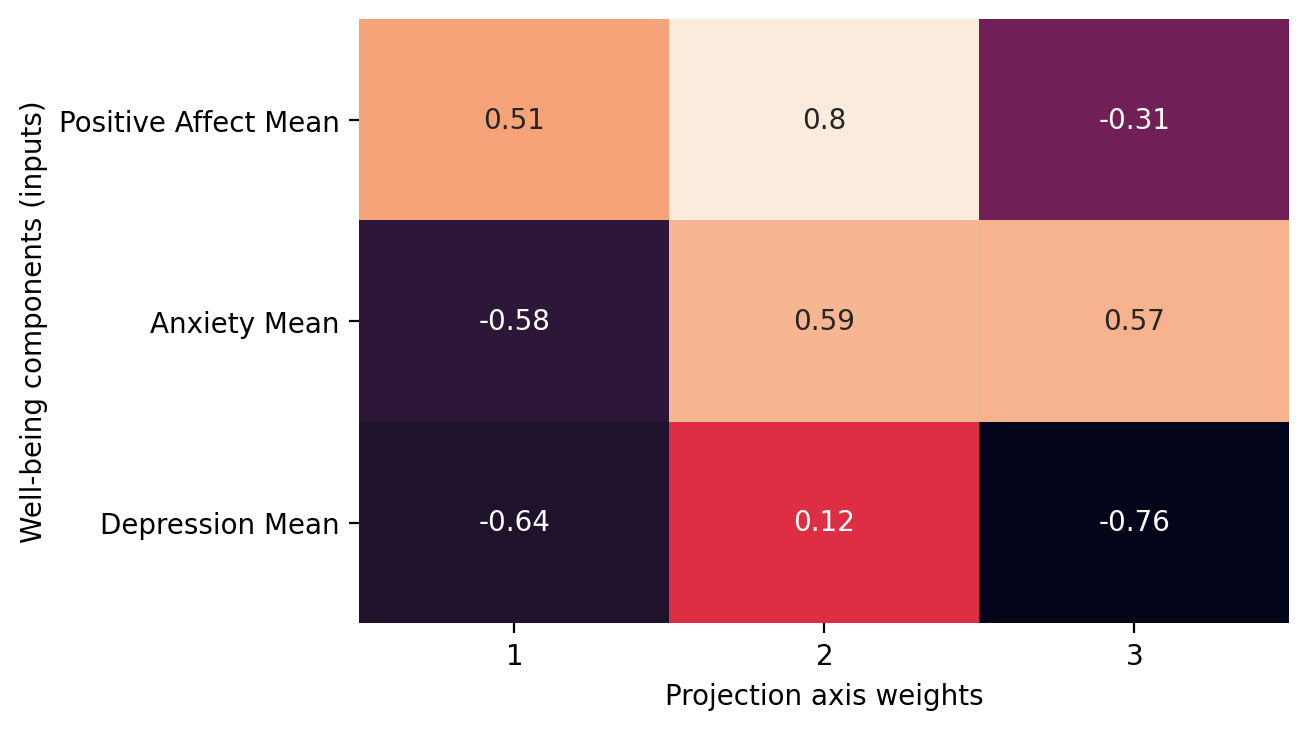


**Supplementary Figure 2**: Results of the principal component analysis run on the student positive affect, depression, and anxiety construct means. The well-being index was constructed from the first principal component. This principal component accounted for more than 50% of the variation seen in the three construct means. This analysis was conducted on the *full dataset* (N = 31981) of students ranging from high school grades 7 to 12, from 2016 to 2019.


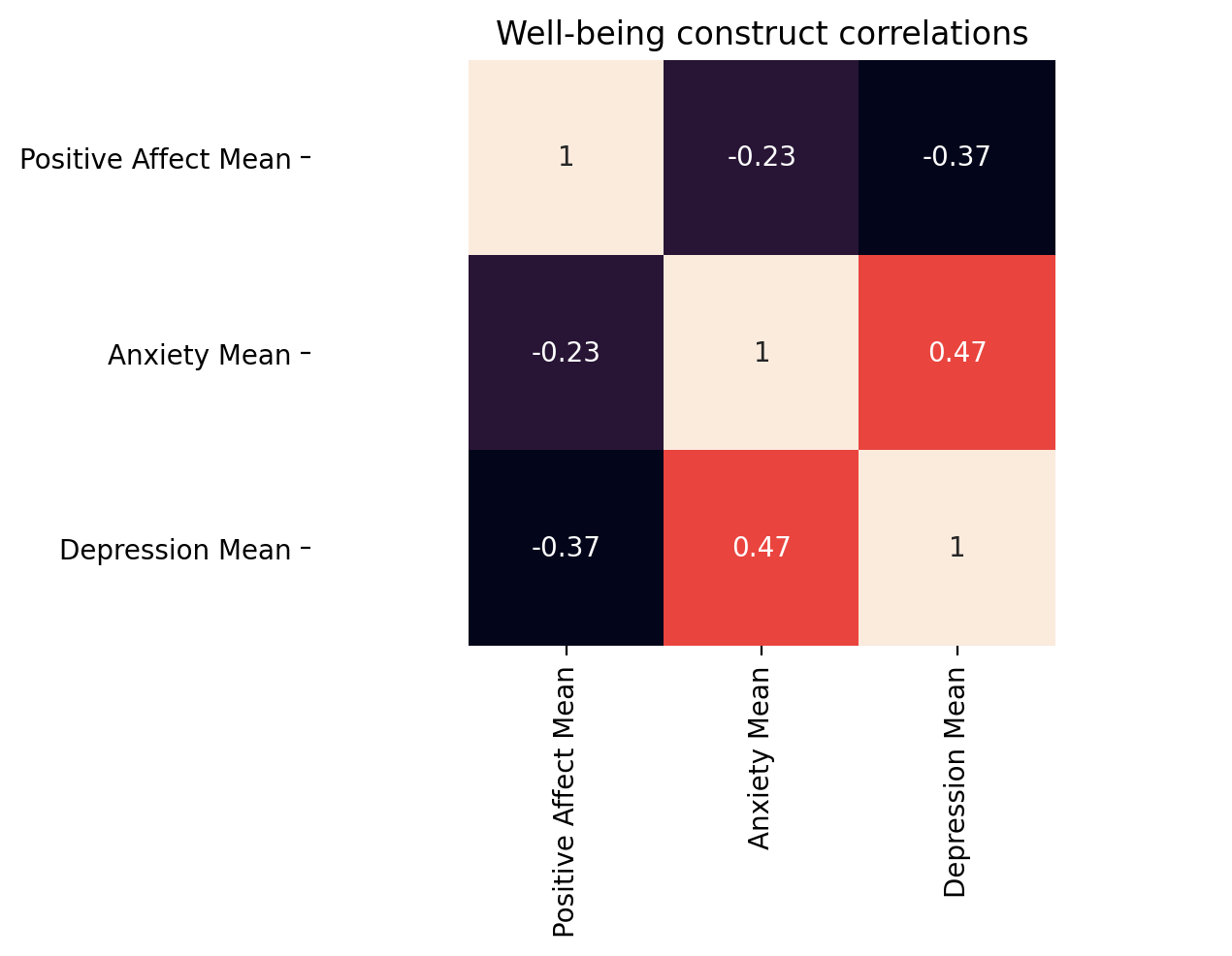


**Supplementary Figure 3**: Correlations between all of the standardised well-being construct means considered in this analysis. These correlations were measured on the *full dataset* (N=31981) of students ranging from high school grades 7 to 12, from 2016 to 2019.
